# Treating Lumbar Fracture Using the Mixed Reality Technique

**DOI:** 10.1155/2021/6620746

**Published:** 2021-03-29

**Authors:** Jiaheng Li, Hexing Zhang, Qiang Li, Shuangqi Yu, Wei Chen, Song Wan, Dong Chen, Rong Liu, Fan Ding

**Affiliations:** ^1^Wuhan University of Science and Technology School of Medicine, Qingling Rd. 1#, Wuhan, 430000 Hubei, China; ^2^Department of Spine Surgery, Wuhan Puren Hospital, Wuhan University of Science and Technology, Benxi Rd. 1#, Wuhan, 430033 Hubei, China; ^3^Institute of Medical Innovation and Transformation, Puren Hospital Affiliated to Wuhan University of Science and Technology, Department of Orthopedics, Puren Hospital Affiliated to Wuhan University of Science and Technology, Benxi Rd. 1#, Wuhan, 430033 Hubei, China

## Abstract

The mixed reality (MR) technique has recently been widely used in the orthopedic surgery with satisfactory results reported. However, few studies have focused on the application of MR in the Lumbar fracture (LF). In this retrospective study, our aim is to analyze some findings by investigating the feasibility of MR applied to lumbar fracture treatment. Posterior vertebrectomy has been operated on 7 patients. The MR–based intraoperative three-dimensional image-guided navigation system (MITINS) was used to assist implantation of pedicle screws. The feasibility and safety of pedicle screw implantation were assessed by postsurgery radiography. The visual analog scale (VAS) and Oswestry Disability Index (ODI) were used to assess the pain level and recovery situation before and after surgery. 57 pedicle screws were safely and precisely placed into three-dimensional lumbar models by using MITINS. No screw was found outside the pedicle of the models, and it was not necessary for the X-ray to provide extra locative information during the operation with the use of MITINS. In summary, the application of MITINS is feasible, safe, and accurate while the lumbar fracture surgery is processing, providing satisfactory assistance for spine surgeons.

## 1. Introduction

With aging of the population, the incidence of osteoporosis has increased significantly [1]. Fractures associated with osteoporosis have become a challenging problem in elderly people. And lumbar fracture is the commonest form of the senile osteoporosis fracture [2]. During the treatment of lumbar fracture, if the fracture is only slightly compressed, conservative treatment or vertebral plasty is usually sufficient. However, if the fracture is severely compressed, or if the type of fracture is burst fracture, open surgery is required [3]. As vertebral degeneration and anatomical variation are very common in the elderly people, the additional problems associated with implanting pedicle screws during an operation are becoming more noteworthy [4].

Recently, digital techniques have been widely applied in orthopedic surgery. With the assistant of clinical real-time position and visualization obtained from such approaches, surgical accuracy has been significantly improved [5]. For example, when we want to improve the safety of lumbar pedicle screw implanting in elderly patients, personalized three-dimensional printing technology makes the implanting of lumbar pedicle screw in patients with severe degeneration or anatomical variation simpler, safer, and more accurate [6]. Computed tomography-guided (CT-guided) navigation and fluoroscopy-based computer navigation systems have also been used to place pedicle screws [7]. Despite these systems that improved the safety and accuracy of the pedicle screw placement [8], the problem of inherent spatial and temporal separation still makes it a tough challenge for spine surgeons. The mixed reality (MR) technique, which is to integrate the real vision and the virtual image, offers a solution that MR can provide efficacy and applicability during the guidance of the surgery, and MR can resolve vision deficiencies during the process of synchronizing virtual images and real operative sights. Our aim was to evaluate a new surgical navigation system based on the MR approach and MITINS while inserting pedicle screws into the elderly patients, which can assess its safety and accuracy during the treatment of lumbar fracture.

## 2. Materials and Methods

### 2.1. Patient Eligibility

From April 2017 to August 2018, 7 lumbar fracture patients who received open surgery by MITINS were selected and assessed retrospectively. Ethical approval and informed data were obtained from each patient. Inclusion criteria were as follows: (1) definitely diagnosed with lumbar fracture, (2) diagnosed with severe decompression and nerve deficiency, (3) over eighteen years old and in full possession of their mental faculties, (4) being able to receive surgery, and (5) MITINS was used during each operation. Excluded criteria were as follows: (1) unable to tolerate surgery, 2) diagnosed with severe diabetes, (3) diagnosed with serious anemia, and (4) diagnosed with severe lung and heart disease.

### 2.2. Operative Strategy and the Rehabilitation Protocol

**Figure 1:**
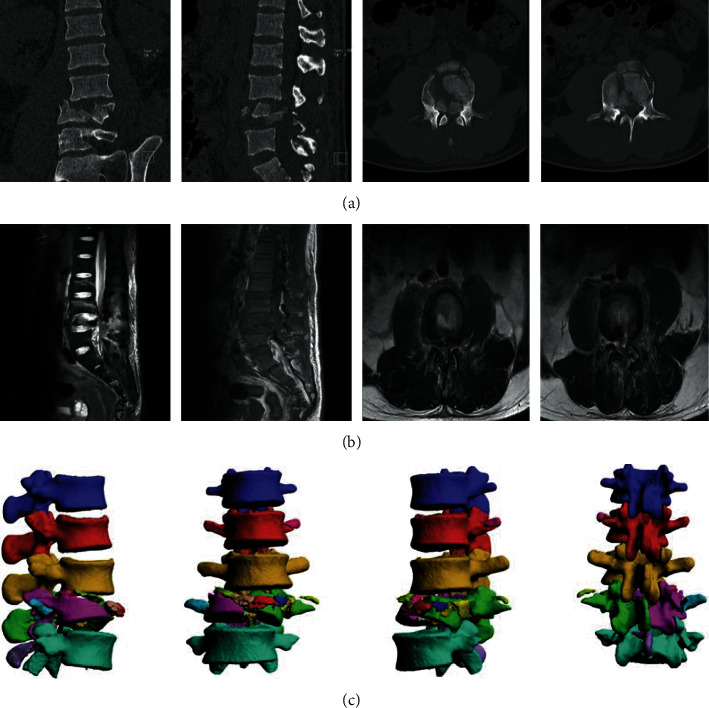
A typical UPA case. A 65-year-old man with LF (L2-L4). (a) CT of lumber presurgery. (b) MRI images of the injured lumbar in the sagittal and the coronal plane. (c) Three dimensional CT images of lumbar.

**Figure 2:**
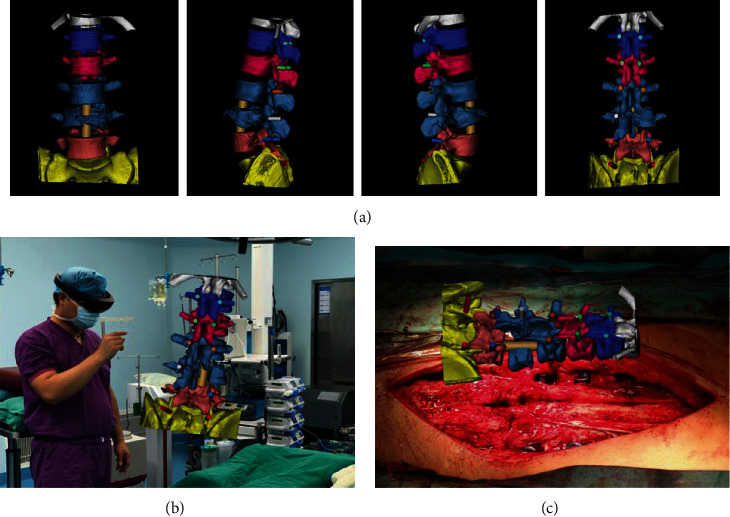
Spine operation of the case identified in Figure 1, performed with the assistance of MITINS. (a) A preoperation surgical demonstration. (b, c) Application of MITINS in the LF operation.

Firstly, fracture-type fixation approaches were analyzed by CT data. Next, the 3D model of the lumbar fracture was constructed, which is built by using MIMICS 10.1 software, then producing 3D printing. Multiple angle views of the lumbar could be seen. A proper stereoscopic and apparent visualization of the lumbar anatomic was completed. We could also achieved an ideal trajectory insertion by eliminating the structural hierarchy. All patients were treated by the same senior surgeon. The operation was performed under general anesthesia. Pedicle screws were inserted into the 3D-printed lumbar model with the assistance of MITINS. Three marks were identified on the preoperative model, and relevant location information was transferred to the computer. The 3D position of the operated vertebrae was determined by three-point registration. By modifying positioning sensors, an optimal registration can be achieved between virtual image and 3D-printed model. Subsequently, the pedicle screws could be placed with the assistant of the MR navigation system from the preoperatively defined ideal pedicle entry point to the terminal point. The subsequent reduction operation procedure was performed as a typical process that a connecting rod was used, and the lateral displacement was relocated by rotating the rod [9]. Anterior dislocation was corrected by lifting the rod, and the vertebral body height was recovered by stretching. Ultimately, the bone graft procedure was completed with titanium cages (Figures 1 and 2).

The rehabilitation protocol was used comprised: 20% mannitol was used for the first 3 days postoperatively, fully drain for 48 hours, followed by removal of the drainage system. The straight leg raising exercise was encouraged after the removal of the drainage system.

### 2.3. Evaluation Index

The accuracy and safety of the pedicle screw insertion were assessed using the postsurgery radiography. The visual analog scale (VAS) and Oswestry Disability Index (ODI) were used to assess the pain level and recovery situation before and after surgery.

### 2.4. Statistical Analysis

Nonparametric data was analyzed using the Wilcoxon rank sum test: *P* < 0.05 was regarded as statistically significant. Statistical Product and Service Solutions (SPSS 15.0.1) software was used for statistical analysis.

### 2.5. Disadvantage of the Application of MR in Lumbar Fracture

However, some limitations of the application of MR in lumbar fracture surgery should be highlighted. First of all, although it can save the expenditure of the complications due to the anatomical and spatial misjudgment of visual limitation of the traditional imaging method, the cost of MR itself is more expensive than the traditional method under the condition of good control of complication after surgery. Secondly, there is a defect of simulation of soft tissues in the MR technique. Thirdly, the effect of navigation during surgery is limited by the factor of light. And different patient postures were needed depending on the operation area, virtual images ,operating table and surgical instruments.

For the surgeon, limitations of MR in lumbar fracture surgery should not be neglected. Firstly, visual discomfort may occur due to the fusion of the real world and the virtual images. Secondly, special training is necessary for the surgeons to tolerate the eye strain induced by the procedure.

## 3. Results and Discussion

**Figure 3:**
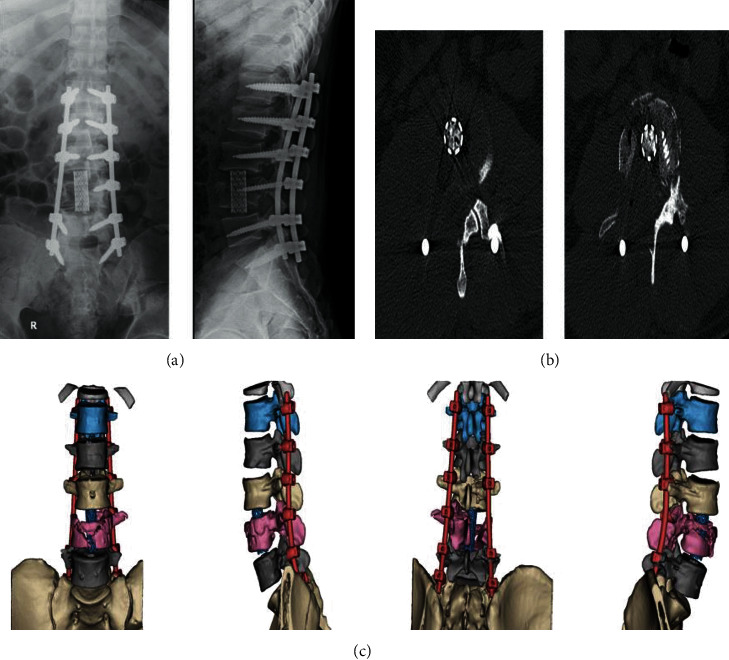
The X-rays and CT results of the abovementioned case identified in Figure 1. (a) X-rays of lumber postsurgery. (b) CT of lumber postsurgery. (c) Three dimensional CT images of lumber postsurgery.

In the present study, 7 patients were received surgery by the assistance of MITINS. After long-term follow-up of the 7 patients, which the period was from 12 to 18 months, the average time is (14.7 ± 1.4) months. 57 pedicle screws (7 patients) were safely and precisely placed into the three-dimensional lumbar models by the assistance of MITINS (Figure 3). No screw was found outside the pedicle of the models, and it was not necessary for the X-ray to provide extra locative information during the operation with the use of MITINS. After surgery, general evaluation of alleviated pain level was assessed by VAS and ODI among all the patients (*P* < 0.05) (Table 1).

With the increase of population, osteoporosis has become an increased dominant disease in the elderly people. It is characterized as a high-risk disease with bone mass decreasing,bone microstructure destroyerd and bone brittleness increasing. [10]. It has been reported that approximately 40% of postmenopausal women are suffering from osteoporotic fractures, and the complications associated with such fractures, including lower limb deep vein thrombosis, bedsores, and urinary tract infections, pose serious threats to the health of the elderly people [11]. Therefore, clarifying the essence and mechanism of fracture cure and carrying out accurate treatment of fractures are major unsolved problems of spine surgery which could produce considerable significance to the society.

Osteoporotic lumbar fracture is very common in the elderly people, and for the lumbar fracture with nerve deficiency, pedicle fixation is widely used. However, due to the serious spine degenerative problem in the elderly people, the consistent and accurate insertion of lumbar pedicle screws i still a challenge for spine surgeons. Compared to the younger group, it is thought that the risk of the failure and nerve injury of posterior pedicle screw placement is significantly higher in the elderly people [12]. However, for burst lumbar fractures and patients with symptoms of severe nerve injury, open surgery still makes an indispensable role for fully decompression of vertebral canal. Pedicle screw placement is also needed to secure the stable internal fixation.

Recently, many ways of inserting pedicle screws in elderly people have been evaluated, including the plate guiding technique, the surgical navigation assistance (SNS), and the surgical robot assistance (SRS) [13, 14]. The plate guiding technique is only suitable for the ordinary lumbar fracture. For burst lumbar fracture, the efficacy of the technique is partial [15]. While SNS and SRS are both feasible methods for spine surgery, there are still some disadvantages. For example, innate waste of manpower and time and extending the duration of operations both make it inevitably difficult to apply to clinical application widely.

The MR-based technique has been used successfully in cervical pedicle screw placement [16]. Preoperative discussion is also an advantage of the MR technique. It can provide the optimized planning of surgical procedures, as its unique displaying approach enables images in the computer to be integrated to the holographic vision which is much closer to the real vertebrae situation [17]. In conventional surgical assistance procedures, such as computer tomography and magnetic resonance imaging, the image data was separated from the surgical site. This brings additional difficulties to spine surgeons to speculate the vertebrae situation, who need connect the information from CT and MRI to the real surgical findings, which requiring strong capacity of visual processing to compensate for the lack of depth perception or the limitation of processing of visualization. Fortunately, using the MR technique, surgeons can easily and effectively acquire good stereoscopic vision in the operation area, instead of shifting their attention to the separate visualization of relevant structures [18]. In the present research, 57 pedicle screws (7 patients) were safely and precisely placed into the lumbar three-dimensional models under MITINS. No screw was found outside the pedicle of the model under the guidance of MITINS. All patients obtained obvious alleviated pain in VAS and ODI after surgery (*P* < 0.05), indicating that the MR technique is feasible, safe, and accurate for the treatment of lumbar fracture.

To the best of our knowledge, this is the first study to report use of the MR technique in lumbar fracture surgery. System components of MITINS consist of trakSTAR,electromagnetic transmitter,and sensors,which is an important support for further combination of deeper obscurez surgery image with more visible and integrated vision. However, some limitations of the application of MR in lumbar fracture surgery should be highlighted. Firstly, special training is necessary for the surgeons to tolerate the eye strain induced by the procedure. Secondly, visual discomfort may occur due to the fusion of the real world and the virtual images. Thirdly, different patient postures were needed depending on the operation area, virtual images, operating table and surgical instruments. Fourthly, this is a small case study that includes no controls.

## 4. Conclusions

The use of MITINS in lumbar fracture surgery is feasible, safe, and accurate, providing satisfactory assistance for spine surgeons.

## Figures and Tables

**Table 1:** VAS and ODI of the included patients.

	VAS	ODI
Presurgery	74.4 ± 4.4	82.7 ± 6.2
3 months after surgery	44.7 ± 4.3	45.7 ± 4.7
6 months after surgery	24.2 ± 2.2	27.4 ± 3.2
12 months after surgery	18.4 ± 2.9	16.4 ± 2.6

## Data Availability

The data used to support the findings of this study are available from the corresponding author upon request.
